# Win or loss? Combination therapy does improve the oncolytic virus therapy to pancreatic cancer

**DOI:** 10.1186/s12935-022-02583-1

**Published:** 2022-04-20

**Authors:** Wenhao Luo, Yawen Wang, Taiping Zhang

**Affiliations:** 1 Department of General Surgery, Peking Union Medical College Hospital, Chinese Academy of Medical Sciences and Peking Union Medical College, No. 1 Shuaifuyuan, Wangfujing Street, Beijing, 100730 China; 2 Department of Endocrinology, State Key Laboratory of Complex Severe and Rare Diseases, Key Laboratory of Endocrinology of National Health Commission of the People’s Republic of China, The Translational Medicine Center of Peking Union Medical College Hospital (PUMCH), PUMCH, Chinese Academy of Medical Sciences and Peking Union Medical College (CAMS & PUMC), Beijing, 100730 China; 3 Clinical Immunology Center, Chinese Academy of Medical Sciences and Peking Union Medical College, Beijing, 100730 China

**Keywords:** Oncolytic virus, Combination therapy, Pancreatic cancer, Novel strategies, Anticancer efficiency

## Abstract

Pancreatic cancer (PC) is a growing global burden, remaining one of the most lethal cancers of the gastrointestinal tract. Moreover, PC is resistant to various treatments such as chemotherapy, radiotherapy, and immunotherapy. New therapies are urgently needed to improve the prognosis of PC. Oncolytic virus (OV) therapy is a promising new treatment option. OV is a genetically modified virus that selectively replicates in tumor cells. It can kill tumor cells without harming normal cells. The activation of tumor-specific T-cells is a unique feature of OV-mediated therapy. However, OV-mediated mono-therapeutic efficacy remains controversial, especially for metastatic or advanced patients who require systemically deliverable therapies. Hence, combination therapies will be critical to improve the therapeutic efficacy of OV-mediated therapy and prevent tumor recurrence. This review aims to investigate novel combinatorial treatments with OV therapy and explore the inner mechanism of those combined therapies, hopefully providing a new direction for a better prognosis of PC.

## Introduction

Pancreatic cancer (PC) is the most lethal malignancy among all cancers, with a 5-year survival rate of 10% [1]. The poor prognosis of PC is mainly attributed to the late symptoms and early metastasis. Currently, radical surgery is the only potential curative way for PC. However, most PC patients were diagnosed late, so no surgery opportunity was left for them. Various cancer treatment options are increasingly developing, such as radiotherapy and immune therapies. But little efficacy was shown in improving patients’ survival. Chemotherapy such as Nab-paclitaxel plus gemcitabine (AG) or FOLFIRINOX, the 1st-line treatment of metastatic PC, can improve the patients’ survival. The median overall survival of AG is 12.1 months, while the median overall survival of FOLFIRINOX is 13.8 months. Although in the current treatment, the survival of patients with PC has been greatly improved, the effect of improving long-term survival is minimal. There is still much room for improvement in the median overall survival of fewer than 2 years. Therefore, new therapies are urgent to be found and applied. Nowadays, novel therapies have been developed for better therapeutic effects against PC, such as oncolytic virotherapy (OVT) [2–4]. To prove the safety and efficacy of OVT monotherapy for PC, pilot studies injected six PC patients with three doses of OV. The results showed no adverse side-effects, but the level of tumor markers decreased and the survival rate increased [5]. OVT uses engineered viruses to destroy cancer cells and activate specific immune responses. Oncolytic viruses (OVs) are cancer therapeutics with multimodal antitumor actions. OVT uses OVs to infect and damage cancerous tissues without damage to normal tissues, because OVs can directly induce cell lysis and systemic antitumor immunity [6]. Because of their capacity to overcome immune escape mechanisms, regulate tumor microenvironment (TME), and promote antitumor immune responses, OVs can become a novel strategy for PC patients. However, OVs treatment has major side effects such as viral infections, and adverse immune-related effects, including fever and rash. Many experts raise the potential safety concerns of OVs. After demonstrating safety in phase I trials, many agents have stepped into phase II efficacy studies, including herpes simplex virus 1, vaccinia virus, adenovirus, parvovirus, and reovirus [7]. For example, a single-arm Phase I trial including 9 patients explored the clinical safety of OVs to PC. The result showed that no patient had adverse effects of OVs, which indicated that OVs were relatively safe for PC treatment [4].

Among PC patients, most treatments are less effective because of the stubborn immunosuppressive microenvironment of PC [8]. OVT can sensitize tumors to immune therapy or chemotherapy. OVs could activate stronger antitumor immunity with strong immunogenic foreign characters, to selectively clear tumor cells without damaging the healthy cells. The combination of chemotherapies and OVs has excellent potential for a profoundly antitumor immune response. Here, we aim to investigate the efficiency of novel combination treatments with OV therapy in PC, hopefully providing a new direction for a better prognosis of PC (Table 1). OVs can be divided into naturally OV and genetically modified viruses. OVs can be genetically modified to selectively target cancer cells or lead to disruptions through antiviral activity. Both OVs types can become therapeutic agents in the treatment of PC. This article discussed different OV types, respectively.

**Table 1:** Novel treatment strategies targeting oncolytic virus combination therapy

Novel strategies	OV types	Characteristics	Combined agent	Function	Refs
H-1PV	Naturally OV	Rodent H-1 protoparvovirus	Gemcitabine/valproic acid/IFN	Elevate macrophage and splenocyte responses against PC cells	[13]
M1 virus	Naturally OV	A strain of alphavirus isolated from culicine mosquitoes	NanoKnife	Electroporation induced by NanoKnife can provide channels for M1 virus by increasing microvessel density and tumor blood vessel permeability	[20]
Oncolytic reovirus	Naturally OV	Oncolytic viruses	CD3-bispecific antibodies	Induce local interferon responses and strong T-cell influx to sensitize the TME for CD3-bsAb therapy	[25, 26]
Pelareorep	Naturally OV	Oncolytic reovirus	Pembrolizumab	Enhancement of PD-L1 in PC after OVT can be antagonized by ICI therapy	[3]
MeV	Naturally OV	Measles vaccine virus	Gemcitabine	Produce great efficacy in treating PC and establishes a novel biological compound to overcome therapeutic resistance of gem to PC	[29, 30]
HF10	Naturally OV	Oncolytic virus derived from a herpes simplex virus-1	Gemcitabine and erlotinib	Induce the PC death and activate anti-tumor immunity	[4]
VSV	Naturally OV	Vesicular stomatitis virus	p53 transgenes	Show stronger replication in virus-resistant PC cells	[35–37]
VSV	Naturally OV	Vesicular stomatitis virus	Polycations and ruxolitinib	Combining polycations with ruxolitinib not only improved overall VSV replication and oncolysis but also accelerated VSV replication	[39–41]
VVL-21	Modified OVs	Oncolytic vaccinia virus with IL-21	α-PD1	Increase the sensitivity of PC to ICI therapy	[47]
CF33	Modified OVs	Chimeric orthopoxvirus	hNIS and anti-PD-L1	Infecting and killing human PCs and producing functional anti-PD-L1 antibody	[48]
OBP-702	Modified OVs	Telomelysin	Chemotherapy, radiotherapy and ICIs	Inhibit the invasion of PC cells via suppression of ERK signaling	[51]
OBP-502	Modified OVs	Telomerase-specific oncolytic adenovirus	ICIs	Induce the release of Immunogenic cell death molecules such as ATP and HMGB1 resulting in recruitment of CD8+ lymphocytes	[54]
AdV or VV	Modified OVs	Oncolytic Adenovirus or vaccinia virus	Vaccination regimen using induced pluripotent stem cells	Provide unique neoantigens by modeling specific epigenetic changes via patient-specific accrual of passenger mutations to activate antitumor immune responses	[57–59]
oVV-Smac	Modified OVs	Oncolytic vaccinia virus expressing Smac	Gemcitabine	oVV-Smac selectively replicates in tumor cells, thus resulting in their lysis, which disrupts the tumor's protection and give gemcitabine an opportunity to penetrate into PC tumor environment	[65]
Ad5	Modified OVs	Adenovirus serotype 5	miR-99b and miR-485	miR-99b and miR-485 function as enhancers of adenoviral oncolysis by improving mature virions	[69]
OAd-TNFa-IL2	Modified OVs	Cytokine-armed oncolytic adenoviruses express TNF-α and IL-2	meso-CAR T cells	Increased both CAR T cell and host T cell infiltration to the tumor with increased TILs	[74–76]
GLV-1h68	Modified OVs	The vaccinia (Lister strain)-derived oncolytic virus	nab-Paclitaxel Plus Gemcitabine	Achieving a virus-mediated induction of a stronger antitumor immunity	[77, 78]
MV-PNP-anti-PSCA	Modified OVs	Measles virus vaccine strains expresses PNP and anti-PSCA	Fludarabine	Double promotors with PNP and anti-PSCA strongly enhanced the oncolytic efficacy of the virus and make PC sensitive to the toxified fludarabine nucleoside analog	[29, 79]
CD/UPRT-armed MDRVV	Modified OVs	MAPK-dependent recombinant vaccinia virus armed with CD an UPRT	5-FC	Armed MDRVV with a suicide gene encoding yeast CD and UPRT, which converts the nontoxic 5-FC into the 5-FU and subsequently into 5-fluorouracil-monophosphate	[81–83]

## Natural OVs

Natural OVs are natural viruses that can kill tumor cells without artificial modification, such as herpes simplex virus, adenovirus, and Coxsackievirus [9].

### H-1 protoparvovirus

The rodent H-1 protoparvovirus (H-1PV) is one kind of OV with natural oncolytic properties,. It can stimulate immune activation in various cancer models [10]. H-1PV-induced oncolysis was shown in various preclinical models of PC [11]. Moreover, H-1PV can become an inducer for chemo-resistance PC because gemcitabine-resistant PC is sensitive to H-1PV [12]. A recent study evaluated the therapeutic efficacy of gemcitabine in combination with H-1PV in the PC model. The combination therapy group showed significant tumor suppression and survival prolongation compared to the gemcitabine monotherapy group [13]. In detail, the effective drug concentration required to inhibit cell proliferation was reduced by up to 15-fold when the cells were infected with H-1PV. H-1PV combined with gemcitabine may be a novel strategy for PC. Another study evaluates the treatment combining H-1PV with the histone deacetylase (HDAC) inhibitor valproic acid (VPA) [14]. HDAC inhibitor can inhibit cancer growth and promote cancer death [15]. VPA can induce death in PC cells when synergized with H-1PV. Mechanically, VPA induces hyperacetylation of viral protein NS1, which enhances H-1PV replication and promotes virus multiplication in tumor cells. Another combination therapy is H-1PV and IFN–mediated therapy. IFN-improves the activation of the virus and inhibits peritoneal carcinomatosis in PC models. Mechanically, H-1PV and IFN- improve animal survival through elevated macrophage and splenocyte responses against PC cells [16].

### NanoKnife with M1 oncolytic virus

NanoKnife is a nonthermal ablation technique for focal tumor ablation in clinical by FDA [17]. M1 virus is a strain of alphavirus isolated from culicine mosquitoes in China [18]. Alpha-virus M1 is a novel natural OV with high tumor selectivity. Combining NanoKnife with the M1 virus could improve the anticancer efficiency in PC. M1 virus can complementally kill the residual cancer cells after NanoKnife, while NanoKnife can enhance M1 virus infection. Electroporation induced by NanoKnife can provide channels for the M1 virus by increasing microvessel density and tumor blood vessel permeability [19]. The size of the channel in the membrane is a few hundred nanometers, much larger than the M1 virus. A recent study showed that NanoKnife combined with the M1 virus inhibits PC cell proliferation. The combination turned immune-silent tumors into immune-activated tumors by T cell activation. Thus, it represents a promising therapeutic efficacy and may improve the prognosis of PC [20]. In conclusion, novel combination therapy of NanoKnife and M1 virus showed a significant synergistic therapeutic efficacy in PC by improving M1 virus infection and T cell activation.

### Oncolytic reovirus converts CD3-bispecific antibody treatment

T-cell-engaging bispecific antibodies can activate profound responses in cancers [21]. CD3-bispecific antibodies (CD3-bsAbs) activate humoral immunity and cellular immunity simultaneously. One functional part of CD3-bsAbs contacts tumor-associated antigen (TAA) from cancer cells, and another part stimulates T cells by CD3 [22]. CD3-bsAbs can induce the polyclonal T-cell pool toward the tumor and reduce the need for endogenous tumor-specific T cells. However, CD3-bsAbs work poorly in tumors with less immune response, such as lack of an IFN gene signature and short of T cells in the tumor beds [23, 24]. OVs can sensitize resistant tumors to immune therapy [25]. A recent study proved that oncolytic reovirus could enhance the efficacy of CD3-bsAbs in immune-silent solid tumors PC [26]. Mechanically, reovirus selectively replicates in tumor cells and confers the PC TME with a strong IFN signature, T-cell-attracting chemokine, increasing NK cells and activated T cells. Oncolytic reovirus induces local interferon responses and substantial T-cell influx to sensitize the TME to CD3-bsAb therapy. Both CD3-bsAbs and OVs undergo rigorous clinical testing and possibly be applied in the clinic. OVs and T-cell-engaging antibody therapy is an emerging and exciting new field of research. The combination of reovirus and CD3-bsAb therapy may become a novel strategy for PC patients.

### Pembrolizumab and oncolytic virus pelareorep

Pelareorep, as oncolytic reovirus, can activate T cell efficacy in PC. Pelareorep treatment has shown reovirus replication, T-cell infiltration, and upregulation of PD-L1. In a follow-up phase 2, single-arm and open-label study, pelareorep combined with gemcitabine showed viral replication and upregulation of PD-L1 in cancer cells of PC patients [27]. A phase 1b study in advanced PC used pelareorep combined with an ICI such as pembrolizumab and chemotherapy including either 5-Fluorouracil, gemcitabine, or irinotecan. These patients were well-tolerated, and the study showed consistent efficacy in patients [3]. Mechanically, PD-L1 will increase after chronic viral infection to minimize immune system-induced damage. However, ICI therapy may antagonize the enhancement of PD-L1 in PC after OVT. ICI therapy plays a significant role in the immune priming effect of OVs in PC. Therefore, combination therapy with pelareorep, pembrolizumab, and chemotherapy is safe and well-tolerated in patients with PC.

### Gemcitabine plus oncolytic measles vaccine virus

Gemcitabine resistance is an obstacle to PC treatment. Measles vaccine virus (MeV) has already shown great oncolytic activity against PC. In preclinical models, MeV has excellent efficacy in treating PC. It establishes a novel biological compound to overcome the therapeutic resistance of PC [28]. In a recent study, MeV could infect and lyse gemcitabine‑resistant PC cells [29]. Another study showed that viral replication is a prerequisite for the effectiveness of oncolysis [30]. Viral replication is crucial for the efficacy and efficiency of OV. Moreover, they found that viral replication cannot be suppressed by gemcitabine. Therefore, the combination of gemcitabine and MeV may become a novel strategy to treat PC.

### HF10 combined with erlotinib and gemcitabine administration

HF10 is derived from a herpes simplex virus-1^50^. It has a potent antitumor effect against PC without damaging normal tissue [31]. As gemcitabine has been well investigated in combination with many OVs in PC, HF10 and gemcitabine could be an ideal therapy against PC with minimal side effects [32, 33]. A phase I trial aimed to evaluate the safety and effectiveness of HF10 combined with erlotinib and gemcitabine in unresectable PC [4]. They found that the antitumor effects of OVs can induce PC death and activate antitumor immunity. Therefore, HF10 combined with erlotinib and gemcitabine was safe and effective for unresectable PC.

### Vesicular stomatitis virus (VSV) based on oncolytic viruses

Vesicular stomatitis virus (VSV) is a promising OV with a solid capacity to replicate in cancer cells. VSV is mainly based on decreased type I interferon (IFN) responses in cancer cells compared to nonmalignant cells [34]. VSV-based OVs are promising strategies against PC. However, some PC cell lines are resistant to VSV. A recent study found novel oncolytic VSVs targeting virus-resistant PC cell lines [35]. Previous studies indicated that p53 variants could be inserted into the VSV genome [36]. A new study established two recombinants, VSV-p53wt and VSV-p53-CC [37]. The functional p53 can enhance these VSV-carried p53 transgenes in cancer cells. In this study, novel oncolytic VSVs show more vigorous replication in virus-resistant PC cells [35]. Future research is urgent to compare the efficacy and safety of the VSV-p53 viruses in vivo. Up to now, we can conclude that VSV-p53 OVs are relatively safe and effective for PC.

Although VSV is effective against PC cell lines, some PC cell lines are still resistant to VSV. JAK1/2 inhibitors such as ruxolitinib can strongly stimulate VSV oncolysis in all resistant cell lines [38]. An early study indicated that different pH conditions or the addition of positively charged polycation, such as Polybrene, could enhance VSV attachment to various cell membrane components by nonspecific electrostatic interactions [39]. A recent study proved that combining VSV with ruxolitinib and Polybrene, which inhibits antiviral signaling, could suppress the resistance of PC cells to VSV. Polycation enhances initial infection, while ruxolitinib strengthens viral replication. They indicated that the resistance of PC to VSV could result from inefficient VSV attachment. Hence, they combined the virus with polycation and ruxolitinib to inhibit the resistance of PC cells to VSV [40]. Mechanically, VSV attachment to PC cells was exceedingly enhanced by polycation. Recent studies reported that Polybrene increased retroviruses’ attachment by tenfold [41]. Combining polycation with ruxolitinib improved VSV replication and oncolysis. Combining VSV with polycation and ruxolitinib can become a novel triple-combination approach to treat PC tumors.

## Modified OVs

Modified OVs are strong antitumor OVs after artificial modification and synthesis, which did not initially have an antitumor function or have a small antitumor function [42].

### Vaccinia virus with IL-21 and Immune checkpoint inhibitors

**Fig. 1:**
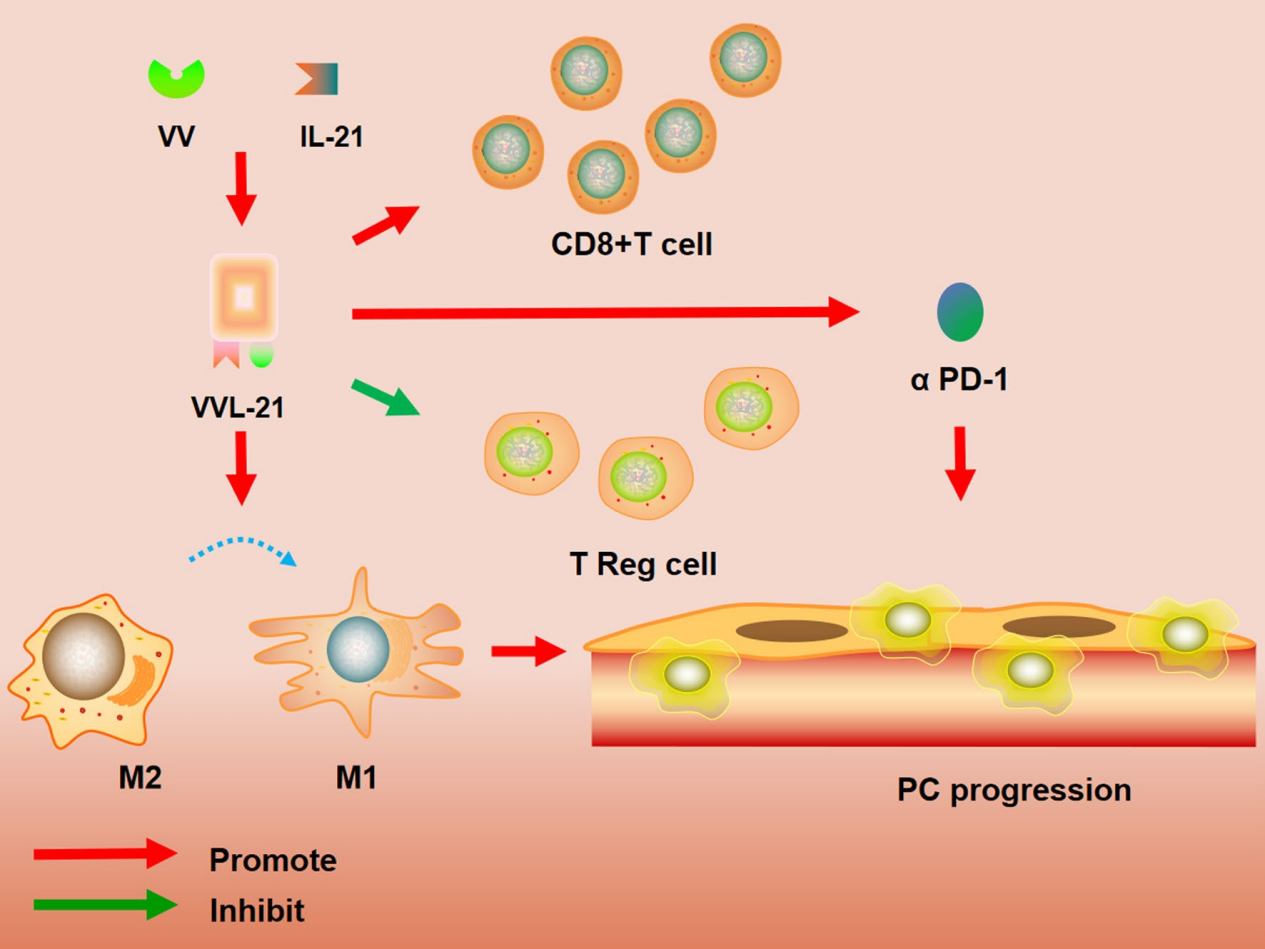
Oncolytic VV with IL-21, namely VVL-21, enhanced stronger antitumor immune responses by repolarizing M2 macrophages to M1 phenotype and encouraged M1 polarization of naïve macrophages8. VVL-21 sensitized PC to the immune checkpoint inhibitors α-PD1

Immune checkpoint inhibitors (ICI) have become a promising novel approach to cancer treatment. However, PC is unresponsive to ICI monotherapy [43]. A recent study proved that OVT could sensitize cancers to ICI therapy and the combination of OVT and ICI treatment has shown increased response rates in many cancers [44]. Another study described the combination of PD1 treatment and OVT [45]. They found that OV-based therapies expand the therapeutic landscape for ICI treatments. Mechanically, OVT may regulate the tumor microenvironment (TME) to induce more robust immune activation and respond more responsive to ICI. Vaccinia virus (VV) is a novel OV to treat PC. A recent study found that a novel VV can attack tumors specifically and activate great anticancer immunity in vivo [46]. The extracellular enveloped virion (EEV) form of the novel VV can avoid clearance by the host immune response, which is the key to efficient cell–cell spread. However, most VV is ineffective because of less EEV. To overcome this obstacle, interleukin-21 (IL-21) is a potent inducer of T cell activation and inhibits the suppressive regulatory T (TReg) cells. A recent study established a new oncolytic VV with IL-21, namely VVL-21, to enhance stronger antitumor immune responses^8^. It repolarized M2 macrophages to the M1 phenotype and promoted M1 polarization of naïve macrophages [47]. Moreover, VVL-21 enhanced adaptive T cell immunity and improved systemic and intra-tumoral effector, memory T cell, and CD8+ T cell populations. They found that VVL-21 sensitized PC to the ICI α-PD1. Based on the remodeling of the immune elements of TME, VVL-21 may increase the sensitivity of PC to ICI therapy. Therefore, the combination of VVL-21 and α-PD1 has better efficacy for PC treatment (Fig. 1).

### CF33-hNIS-anti PDL1

OVs and ICIs are suggested to treat PC because OVs can sensitize tumors to ICIs therapy [44]. CF33 is armed with the human sodium iodide symporter (hNIS) and anti-PD-L1 antibody. It targets cancer cells and produces the anti-PD-L1 protein, which could further enhance antitumor immune cell function [44]. CF33-hNIS-antiPDL1 is a genetically engineered chimeric orthopoxvirus. A recent study shows that CF33- hNIS-antiPDL1 has oncolytic efficacy against human PC cell lines and further confirms its safety [48]. Mechanically, the CF33-hNIS-antiPDL1 virus functions properly in oncolytic efficacy by effectively infecting, killing human PC and producing functional anti-PD-L1 antibodies. Intraperitoneal delivery of CF33-hNIS-antiPDL1 reduces the peritoneal tumor burden and improves survival. Therefore, CF33-hNIS-anti PDL1 can become a novel strategy to treat PC.

### OBP-702

The gene modification in telomerase-specific virus OBP-301 (telomelysin) enables OBP-301 to replicate selectively in tumor cells and induce tumor-specific oncolytic cell death. As oncolytic virotherapy, the telomerase-specific virus OBP-301 exhibits broad-spectrum antitumor effects against various cancers, including PC [49]. OBP-702 is a modified OBP-301 that can induce the tumor suppressor gene p53 by gene modification. Through activating p53, OBP-702 exhibited greater antitumor efficacy than OBP-301. Moreover, OBP-702 has therapeutic potential against various p53-inactivated cancers, including PC [50]. A recent study indicates that OBP-301 and OBP-702 inhibit PC development by inducing autophagy and apoptosis. In addition, the OBP-702 can activate p53, while OBP-301 does not activate p53 in PC. Therefore, OBP-702, p53-armed oncolytic virotherapy, could be a promising strategy for PC [51]. Mechanically, OBP-702 effectively inhibited the invasion of PC cells by inhibiting ERK signaling. OBP-702 combined with chemotherapy, radiotherapy, and ICIs is underway for PC treatment. Further clinical studies are needed to confirm the safety and feasibility of OBP-702 in invasive PC.

### Telomerase-specific oncolytic adenovirus and anti-PD-1

Immunogenic cell death (ICD) can induce an effective antitumor immune response by activating dendritic cells (DCs) and T lymphocytes. ICD is characterized by the secretion of damage-associated molecular patterns (DAMPs), such as high-mobility group box protein 1 (HMGB1) and adenosine triphosphate [52]. ICI monotherapy has limited efficacy for PC patients. OVs have been reported to induce ICD by combining with ICIs [53]. A recent study found that OBP-502, an OBP-301 variant, can induce ICD and increase the efficacy of PD-1 Ab in PC. Mechanically, OBP-502 significantly increased the release of ATP and HMGB1 by inducing autophagic and apoptotic cell death of PC. In detail, OBP-502 induced the release of ICD molecules such as ATP and HMGB1, leading to the recruitment of CD8+ lymphocytes. Combination therapy of OBP-502 and PD-1 Ab inhibited the growth of PC by recruiting CD8+ lymphocytes [54]. Therefore, OBP-502 combined with PD-1 could turn immunologically “cold” tumors into “hot” tumors. Therefore novel oncolytic virotherapy is an ideal inducer for ICIs. The combination therapy can improve clinical benefits for patients with PC.

### OV and vaccination

A recent study showed that oncolytic AdV or VV could broadly induce tumor-specific immunity. They develop a novel vaccination regimen using induced pluripotent stem cells (iPSC), gene editing, and tumor-targeted replicating oncolytic viruses^35^. The study found that tumor-specific solid T-cell responses are well-tolerated and nontoxic [55]. Another study showed that AdV and VV are effective for eliminating of PC in vivo [56]. The adjuvant value of OV was proved by infecting autologous tumor cells with replicating AdV or VV, and transporting these cells. They created a somatic cell-derived tumor cell vaccination by combining OV with the iPSC technology platform. The mechanism of the method is patient-matched iPSC technology. It provides unique neoantigens by modeling specific epigenetic changes through the accrual of passenger mutations [57]. Moreover, OV can activate antitumor immune responses, which induces virus-induced ICD. Indeed, they found that both AdV and VV can cause ICD. In detail, AdV is effective at TLR activation, resulting in early T-cell activation and Treg suppression [58]. VV expresses immune-modulatory proteins that may promote activated T-cell responses or downregulate effective immune responses before using AdV [59]. Therefore, the sequential use of two distinct OV can effectively prevent PC progression.

### Gemcitabine and engineered OV

**Fig. 2:**
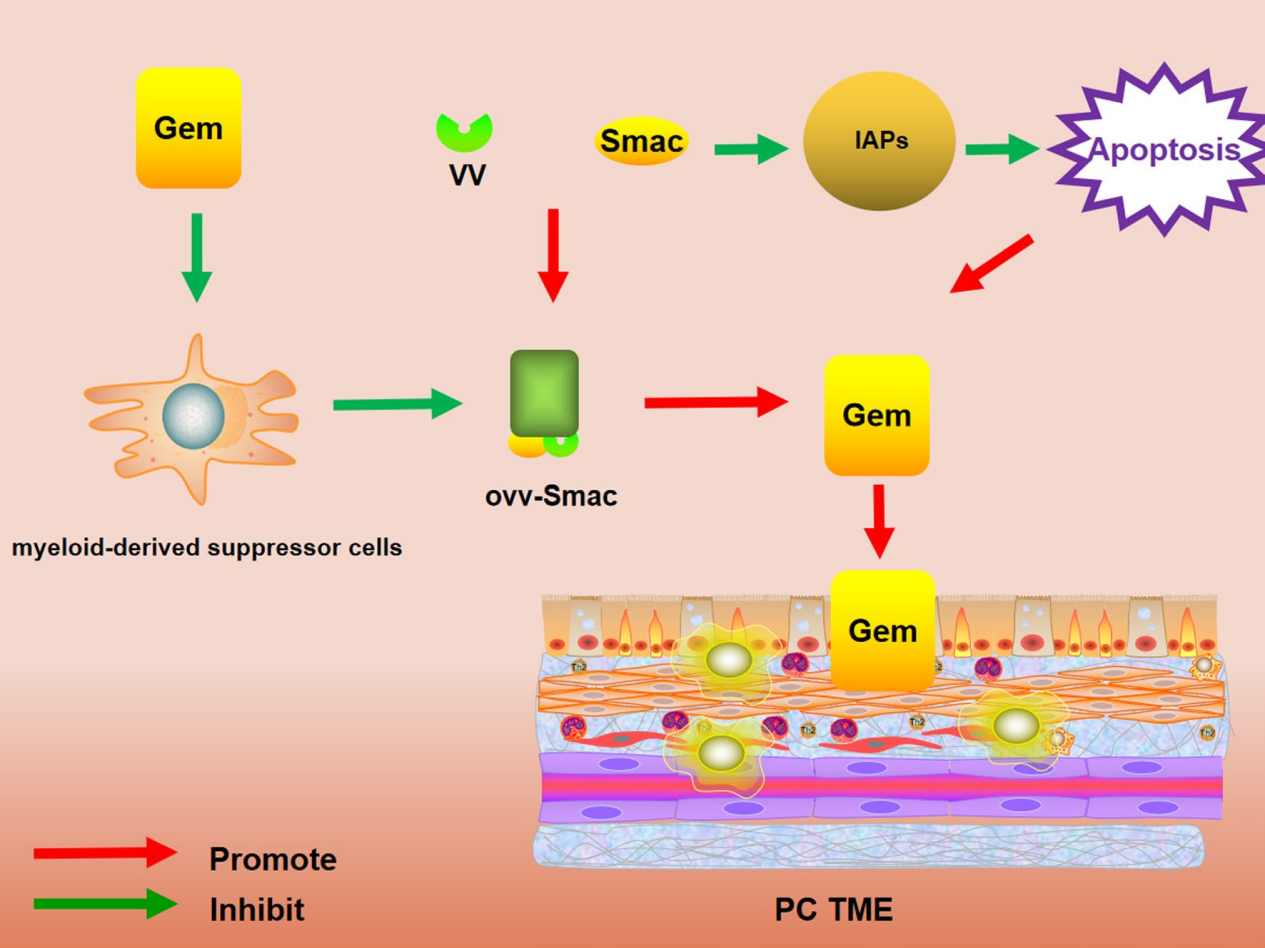
oVV‑Smac selectively replicates in tumor cells, thus resulting in their lysis, disrupting the tumor's protection and allowing gemcitabine to penetrate the PC tumor environment. Gemcitabine can enhance vaccine efficacy by eliminating myeloid‑derived suppressor cells in PC. Both oVV‑Smac and gemcitabine induce apoptosis, which results from the synergistic effects

VV with strong immunogenic has become ideal oncolytic immunotherapy [60]. VV is a promising novel therapy for cancer treatment [61]. VV‑based gene therapy has been investigated in PC and could inhibit significant PC growth with relatively limited side effects [62]. The second mitochondrial‑derived activator of caspase (Smac) is produced during apoptosis. A study showed that Smac could activate apoptosis in PC by inhibiting the inhibitor of apoptosis proteins (IAPs) [63]. Smac was proved to enhance the sensitivity to chemotherapy. It indicated that the targeted delivery of Smac may be a promising gene therapy in PC [64]. Based on the studies above, regulating Smac expression seems a promising therapy in PC. Oncolytic VV expressing Smac (oVV‑Smac) combined with gemcitabine in PC has been researched in vitro and vivo. The results showed Smac exhibited low expression in PC cell lines. Moreover, co‑treatment with oVV‑Smac and gemcitabine resulted in a synergistic effect [65]. Mechanically, oVV‑Smac replicates selectively in tumor cells, leading to their lysis, disrupting the tumor's protection and allowing gemcitabine to penetrate the PC tumor environment. Furthermore, gemcitabine enhanced vaccine efficacy by eliminating myeloid‑derived suppressor cells in PC. Gemcitabine may stimulate the viral uptake in PC cells. In addition, oVV‑Smac and gemcitabine induce apoptosis by synergistic effects [66]. Therefore, these findings indicate the sound effects of co‑treatment with oVV‑Smac and gemcitabine (Fig. 2).

### miR-99b, miR-485, and modified adenoviral oncolysis

Oncolytic adenoviruses are therapeutics under clinical development. It would be feasible to re-establish genes by specific miRNAs for productive viral infection and improving adenoviral oncolysis. Adenovirus serotype 5 (Ad5) is an oncolytic agent for treating various malignancies, including PC [67]. After adenoviral infection, cellular miRNAs have significant changes influencing viral replication [68]. These changes in miRNAs may generate an inadequate environment for adenoviral activity. A recent study identified a group of miRNAs that sensitize cells to Ad5-induced cell death. However, only miR-26b was an enhancer of Ad5 propagation [68].

A recent study showed that miR-99b and miR-485 could improve mature virions as enhancers of adenoviral oncolysis. Increased adenoviral activity may result from increased E1A and late viral protein expression. Mechanically, inhibition of the transcriptional repressors ELF4, MDM2, and KLF8, miR-99b or miR-485 target genes, can promote the viral protein expression. Arming the oncolytic adenovirus with miR-99b or miR-48 can strengthen the antitumoral activity. The results demonstrate that miR-99b and miR-485 are sensitizers of adenoviral replication. miR-99b and miR-485 led to the earlier release of infective virions, enhancing lytic effects. miR-485 expression increases to reduce RIG-1 levels and diminish the induction of downstream antiviral proteins [69]. miR-99b facilitates hepatitis B virus replication by increasing autophagic activity and promoting HBV protein production [70]. Thus, miR-99b and miR-485 might have a proviral function. Moreover, the infection of PC cells with AdwtE miR-99b and AdwtE miR-485 was associated with higher expression of viral genes encoding structural proteins and the E1A gene. It suggests that the miR-99b and miR-485 effects were closely associated with the transcriptional activation of viral genes. Thus, we propose that the downregulation of KLF8, ELF4, and MDM2 through miR-99b or miR-485 promotes the adenoviral late genes and E1A, which facilitates the adenoviral life cycle. The expression of miR-99b and miR-485 may provide PC with tumor suppressor activities. Furthermore, they also found that miR-99b and miR-485 were poorly expressed in PC samples. The poor expression was related to poorer survival rates [71]. Therefore, AdwtE miR-99b or AdwtE miR-485 may become a novel strategy for treating PC.

### Cytokine-armed oncolytic adenoviruses (AdV)

PC has few tumor-infiltrating lymphocytes (TILs). The lymphocytic populations are mainly in the surrounding of PC. A recent study indicated that the T cells in PC patients target neoantigens [72]. Another study showed that AdV-TNFa-IL2 could strongly induce robust chimeric antigen receptor T (CAR T) cell infiltration, thus enhancing antitumor efficacy. Respectively, TNF-α can induce T cell–attractive chemokines [73], and IL-2 can activate the proliferation of T cells [74]. CAR T cell therapy has efficacy in lymphoblastic leukemia. For CAR T cell therapy, mesothelin is a crucial target overexpressed in PC [75]. Mesothelin redirected chimeric antigen receptor T cell (meso-CAR T cell) therapy has shown practical efficacy in clinical trials. In general, the primary mechanism of cytokine-armed AdV plus CAR T cell therapy was related to enhanced T cell function in the tumor. A recent study showed that combined meso-CAR T cells with an oncolytic adenovirus expressing TNF-α and IL-2 would improve efficacy. They engineered AdV to express TNF-α and IL-2 and combine it with CAR T cells targeting mesothelin. AdV-TNFa-IL2 strengthens the antitumor efficacy of meso-CAR T cells with increased TILs and prolonged T cell function in PC. They also found significant tumor regression in mice engrafted with immunosuppressive PC. AdV-mTNFa-mIL2 increased the tumor’s CAR T cell and host T cell infiltration. These outcomes showed that combining cytokine-armed AdV could overcome the immunosuppressive TME [76]. AdV could increase the efficacy of CAR T cell therapy. Combined oncolytic adenovirus expressing TNF-α and IL-2 with meso-CAR T cells is a novel combination therapy in PC treatment. The combination therapy increases the efficacy by regulating the immunosuppressive TME and enhancing immunities.

### GLV-1h68 with nab-paclitaxel plus gemcitabine

The vaccinia (Lister strain)-derived oncolytic virus GLV-1h68 was produced by inserting special expression cassettes encoding at gene loci of the virus [77]. A recent study evaluated the efficacy of GLV-1h68 combined with nab-paclitaxel and gemcitabine in PC cell lines^65^. They found that the combination of GLV-1h68 with chemotherapies was effective in PC^65^. It is feasible to achieve stronger antitumor immunity mediated by the virus [78]. We found that triple combination therapy has excellent efficacy in PC cell killing. Hence, combination GLV-1h68 plus nab-PTX plus gemcitabine may become a novel therapeutic strategy for PC treatment in the future.

### Modified MV and GEM

Measles virus (MV) vaccine strains have significant oncolytic activity targeting malignancies. Viruses have been modified to express suicide genes, thus increasing oncolytic efficacy. *Escherichia coli* purine nucleoside phosphorylase (PNP) converts fludarabine to 2-fluoroadenine and increases MV oncolysis. 2-Fluoroadenine can be metabolized to toxic ATP analogs that inhibit DNA, RNA, and protein synthesis. PNP is important for fludarabine. Therefore, fludarabine combined with MV-encoded PNP can enhance its therapeutic efficacy. A recent study established a new MV that enters cells by prostate stem cell antigen (PSCA), expressed on PC rather than normal tissue [79]. PSCA expression levels differ between PC and normal tissues. It is feasible to enter through PSCA selectively. The novel MV is MV-PNP-anti-PSCA, which expresses PNP. Double promotors with PNP and anti-PSCA strongly enhanced the oncolytic efficacy of the virus [29]. MV-mediated PNP and fludarabine strategy in PC models had a profound efficacy. Moreover, various PC cell lines were still sensitive to the fludarabine nucleoside analog with MV-PNP-anti-PSCA. In conclusion, the effect of oncolytic MV can be enhanced by PNP/fludarabine system for PC therapy.

### CD/UPRT-armed MDRVV

VV is a double-stranded DNA virus of the family Poxviridae. A recent study showed that VV could kill cancer cells by lysis and triggering antitumor immune responses [80]. Novel strategies focus on the genetic modification of the VV genome. Pexa-Vec (JX-594), a new VV immunotherapeutic agent, deleted the viral gene that encodes thymidine kinase and granulocyte/macrophage-colony-stimulating factor (GM-CSF)^68^. It could enhance antitumor immune responses by dendritic cell and T cells activation [81]. Erlotinib and selumetinib are therapeutic agents targeting the MAPK pathway for PC. MAPK-dependent recombinant vaccinia virus (MDRVV) would be more effective in tumor cells. A recent study showed that MDRVV with a suicide gene encoding yeast cytosine deaminase (CD) and uracil phosphoribosyltransferase (UPRT) converts the nontoxic 5-fluorocytosine (5-FC) into the 5-fluorouracil (5-FU) and subsequently into 5-fluorouracil-monophosphate [82]. Another study evaluated MDRVV with a bifunctional fusion gene encoding CD/UPRT. They found that CD/UPRT-armed MDRVV could efficiently eliminate PC and were enhanced with 5-FC. These findings indicated that MDRVV armed with CD/UPRT and combined with 5-FC is a promising therapeutic strategy for PC [83]. CD/UPRT-armed MDRVV can broadly inhibit PC, and combining it with 5-FC can increase its efficacy. Therefore, we strongly suggest that MDRVV armed with CD/UPRT and combined with 5-FC is a promising strategy for PDAC treatment.

## Conclusion and future perspectives

This article found that OV treatment has been widely assessed by clinical trials against PC. However, OVs monotherapy remained a significant challenge to treat PC fully. Increasing clinical outcomes proved the efficacy of combination therapy with OVs in PC. Hence, future research should establish novel OVs or potential combination treatment methods specific to PC.

Increasing studies proved the PC killing capacity of virus infections. Up to now, we have found various OVs with strong antitumor efficacy due to stimulating apoptosis and enhancing immune attacks against PC. Although recent studies proved the safety of various viruses, controlling side effects remained an important challenge of oncolytic virus anti-cancer therapy. Moreover, the disadvantages of OV included difficult administration of OVs because of the pre-existing neutralizing antibodies, innate immune response and poor targeting delivery efficacy. Moreover, monotherapy of OVT can indeed improve the efficacy of PC treatment, but it is too moderate to reach a reasonable outcome. To overcome these obstacles, combined therapy with OVT could fully meet the clinical requirements [84].

A lot of systemic delivery models have been developed to improve the possibility, safety, and efficiency of OVT and help OVs overcome biological delivery obstacles of the PC tumor microenvironment. The preclinical and clinical data above showed that combined traditional therapies with OVT have excellent potential to improve PC treatments. They will become future directions of PC novel immune therapy methods. In summary, OV can efficiently be engineered for targeted therapy of PC. Arming novel agents or combining novel strategies can enhance the oncolytic effect significantly. We suggest that OV combination therapies are safe and effective. In the future, combination therapy should be explored further in extensive prospective studies and be applied in clinics as soon as possible.

## Data Availability

Not applicable.
